# Predictors of the Development of Gastric Cancer in Post-*Helicobacter pylori*-Eradication Patients Followed Up for More than 10 Years: A Histological, Serological, and Endoscopic Study

**DOI:** 10.3390/cancers17030552

**Published:** 2025-02-06

**Authors:** Kazuhiro Mizukami, Masaaki Kodama, Yuka Hirashita, Masahide Fukuda, Sotaro Ozaka, Koshiro Tsutsumi, Ryota Sagami, Kensuke Fukuda, Ryo Ogawa, Kazunari Murakami

**Affiliations:** 1 Department of Gastroenterology, Faculty of Medicine, Oita University, 1-1, Idaigaoka, Hasama, Yufu 879-5593, Japanmurakam@oita-u.ac.jp (K.M.); 2 Department of Advanced Medical Sciences, Faculty of Medicine, Oita University, 1-1, Idaigaoka, Hasama, Yufu 879-5593, Japan

**Keywords:** *Helicobacter pylori*, gastric cancer, eradication, serum pepsinogen, endoscopic atrophy, long-term follow-up

## Abstract

Background/Objectives: Although *Helicobacter pylori* (*H. pylori*) eradication therapy is important for preventing gastric cancer (GC), the occurrence of GC after *H. pylori* eradication remains a problem. In this study, the aim was to identify risk factors for GC after *H. pylori* eradication by comparing long-term histological, endoscopic, and serological evaluations of patients with and without GC. Methods: Patients who underwent *H. pylori* eradication therapy at Oita University Hospital between June 1997 and August 2013 and were followed for at least 3 years with long-term endoscopy, histology, and serum biochemical tests were included, and the GC (215 cases) and non-GC (11 cases) groups were compared. Results: The GC group was older than the non-GC group at the time of eradication, had lower serum pepsinogen I/II levels, had severe endoscopic atrophic changes, had higher activity at the antrum, and inflammation and intestinal metaplasia (IM) at the corpus on updated Sydney system scoring. On long-term follow-up after eradication, the GC group had a wider range of endoscopic mucosal atrophy and a lower serum pepsinogen I/II ratio at any time point. Conclusions: Endoscopic mucosal atrophy and the serum pepsinogen I/II ratio are useful predictors of GC in patients post *H. pylori* eradication at any time point.

## Simple Summary

The aim of our study was to compare the groups of patients who had developed gastric cancer (GC) and those who had not, among those who had been able to be observed for more than 3 years after *Helicobacter pylori* eradication, and to identify risk factors that make it more likely to develop GC after eradication. Comparing the endoscopic, histological, and serum biochemical findings of the cases, we found that GC developed in cases where the patient was older at the time of eradication had lower serum pepsinogen I/II levels, had more severe endoscopic atrophy, had higher activity at the antrum, and inflammation and intestinal metaplasia at the corpus. Furthermore, in the long-term follow-up, endoscopic mucosal atrophy was severe, and serum pepsinogen I/II levels were low at all points in the GC group; these could be useful factors for predicting the occurrence of GC post *Helicobacter pylori*.

## 1. Introduction

Although *Helicobacter pylori* (*H. pylori*) infection has been identified as the cause of gastric cancer (GC) [1,2], it remains one of the diseases that urgently need to be addressed regarding diagnosis and treatment, since GC is responsible for the sixth highest number of cancer cases and accounts for the fourth highest number of deaths from cancer, with 1.09 million cases of death due to GC in the world in 2020 [3]. Although numerous reports have shown that *H. pylori* eradication therapy prevents GC [4,5], it does not prevent it completely, with a risk ratio of approximately 0.34–0.54 [5,6,7]. Therefore, patients should undergo endoscopic screening to prevent death from GC even after *H. pylori* has been eradicated [8].

In recent years, the number of GC cases detected after *H. pylori* eradication has increased. About the cause of GC after eradication, involving the possibility that cancer has already developed as occult cancer at the time of eradication is considered, but it has been reported that, due to changes in the gastric environment after eradication, the clinicopathological characteristics of post-eradication GC differ from those of *H. pylori*-positive GC [9,10], and this is still under discussion.

Changes in serum biochemical findings can also be observed after *H. pylori* eradication. Pepsinogen (PG) is an important serum marker for atrophic gastritis, a precancerous change in GC. PG can be classified as PG I and PG II. PG I is produced by the chief and mucous neck cells in the fundic glands, whereas PG II is produced by these cells and by the cells in the pyloric glands and Brunner’s glands [11]. The “PG method” focuses on the relationship between PG and gastric mucosal atrophy [11]. Hanada et al. showed that the PG I/II ≤ 4.5 group after eradication had a higher risk of developing GC than the PG I/II > 4.5 group [12]. Miki et al. proposed a GC risk screening method using the “ABC method”, a combined test of serum anti-*H. pylori* IgG and serum PG levels [11], and Yoshida et al. followed groups stratified by the ABC method for 16 years and found that the incidence and hazard ratio of GC increased significantly and progressively with the progression of atrophy [13].

We have reported endoscopic, histological, and gastric mucosal changes in patients more than 10 years after successful eradication of *H. pylori* [14,15]. As serological changes, anti-*H. pylori* antibodies, PGI, II, and I/II ratios were reported for 10 years after eradication [16]. Furthermore, we analyzed the changes in anti-*H. pylori* antibodies and anti-*CagA* antibodies after eradication, and we reported differences in antibody titers in the post-eradication GC risk group [17]. However, there have been no reports to date examining the risk of GC after eradication in patients followed for more than 10 years after *H. pylori* eradication. In the present study, the long-term trends in histological evaluation and serological tests in post-eradication GC patients and post-eradication non-GC patients were examined, and risk factors for GC after *H. pylori* eradication were investigated to facilitate efficient follow-up and screening.

## 2. Materials and Methods

### 2.1. Participants

Of the 2876 patients who successfully completed *H. pylori* eradication therapy at Oita University Hospital between June 1997 and August 2013, those who could be followed-up every one or two years by upper gastrointestinal endoscopy, histological evaluation, and serological evaluation were included. The following patients were excluded: (1) patients younger than 20 years; (2) cases of unsuccessful eradication or re-infection of *H. pylori*; and (3) patients who had been followed up for less than 3 years. Patients whose histological or serological evaluations were interrupted during the follow-up period were also excluded. The study protocol was approved by an institutional review board of Oita University, Faculty of Medicine (Approval No. 2339, approved on 25 July 2022). All procedures followed were in accordance with the ethical standards of the responsible committee on human experimentation (institutional and national) and with the Helsinki Declaration of 1964 and later versions. Informed consent or a substitute for it was obtained from all patients for inclusion in the study.

### 2.2. Study Design

This was a single-center, retrospective, case–control study. All patients were divided into two groups: a GC group who developed GC during follow-up; and a non-GC group who did not develop GC. All GC patients were diagnosed at Oita University Hospital, and the final diagnosis was obtained from resected specimens, either endoscopically or surgically. All patient information was collected from the medical information in the electronic medical record, and the following information was obtained: sex, age at *H. pylori* eradication, period between eradication and development of GC, average observation period after eradication, and background of the stomach. Patient background characteristics, serum pepsinogen (PG) levels, extent of endoscopic mucosal atrophy, and histological evaluation at the time of eradication were compared between the GC group and the non-GC group. Changes over time in serum pepsinogen (PG) levels, endoscopic mucosal atrophy, and histological findings were also compared between the GC group and the non-GC group, and risk factors for the development of GC after *H. pylori* eradication were investigated.

### 2.3. Helicobacter pylori

*H. pylori* infection status was determined by tissue, culture, and the rapid urease test (RUT), and a positive result for any of these was defined as current infection. Successful eradication was confirmed as negative results for the RUT, histology, culture testing, and the urea breath test. These tests for *H. pylori* infection status were confirmed repeatedly, along with histological and serological evaluations during follow-up.

### 2.4. Endoscopic Findings

Endoscopic examinations were performed using high-performance endoscopes (Q-260, HQ-260, and HQ-290 and others, Olympus, Tokyo, Japan and EG-L590ZW, Fujifilm Co., Tokyo, Japan). Endoscopic findings of gastric mucosal atrophy were assessed according to the Kimura–Takemoto classification [18], which defines atrophic patterns based on the following characteristic features: C1, when atrophic mucosa is only found in the antrum; C2, when atrophic mucosa is found at the gastric angle or in the lower corpus; C3, when atrophic mucosa is also found in the upper corpus; O1, when atrophic mucosa surrounds the gastric cardia, but the folds of the greater curvature are relatively maintained; O3, when the entire gastric mucosa is atrophic, and the folds of the greater curvature as a whole have disappeared; and O2, representing an intermediate condition between O1 and O3. In this study, the endoscopic atrophy score was defined as follows: absence of any atrophy, 0; C1, 1; C2, 2; C3, 3; O1, 4; O2, 5; and O3, 6.

### 2.5. Histology

During annual endoscopic examinations, biopsy specimens were taken from two gastric mucosal sites: the greater curvature of the antrum, 2–3 cm from the pylorus; and ~8 cm from the cardia of the greater curvature of the corpus. These sites are among the five biopsy sites recommended in the updated Sydney system [19]. The specimens were fixed with 10% buffered formalin for 24 h and embedded in paraffin. Vertical serial sections were stained with hematoxylin and eosin.

The grade of gastric inflammation in each specimen was evaluated using the analog scale of the updated Sydney system. Mononuclear cells, neutrophils, atrophy, and intestinal metaplasia (IM) were classified as: 0, ‘normal’; 1, ‘mild’; 2, ‘moderate’; and 3, ‘marked’. Histological evaluations were performed by an experienced pathologist from Oita University Hospital.

### 2.6. Serum Pepsinogen Evaluation

Serum samples were collected in a fasting state prior to endoscopy and stored at −80 °C until assayed in the laboratory. Serum PG values were measured by the CLIA method (ARCHITECT pepsinogen I, II; Abbott Japan LLC, Tokyo, Japan) until March 2012, after which serum PG values were measured by latex agglutination (LZ test “Eiken” pepsinogen I, II; Eiken Chemical, Tokyo, Japan). Serum PG has attracted attention as a factor that predicts the degree of gastric mucosal atrophy and defines the risk of GC development [20,21], and patients with PG I ≤ 70 ng/mL and PG I/II ≤ 3 are considered a high-risk group for GC. In this study, the mean values of PG I, PG II, and PG I/II were calculated for both groups and compared.

### 2.7. Endpoints

The primary endpoint was the identification of factors contributing to the development of GC during long-term follow-up in patients after *H. pylori* eradication. Secondary endpoints included comparisons of changes in patient background and status, serum PG levels, endoscopic mucosal atrophy, and gastric histology between the GC and non-GC groups.

### 2.8. Statistical Analysis

Statistical analyses were performed using SPSS software (SPSS Statistics 28, SPSS, Tokyo, Japan), and data are expressed as mean ± standard deviation (SD). Fisher’s exact test was used to compare sex between groups. Student’s *t*-test was used to compare groups in terms of serum pepsinogen levels. Mann–Whitney U test was used to compare endoscopic gastric atrophy scores and updated Sydney system scores between the groups. *p*-values less than 0.05 were considered significant. In addition, propensity score matching was used to adjust for the involvement of confounding factors in the comparison of GC and non-GC groups. Sex- and age-matched GC and non-GC cases were selected as the matched GC group and matched non-GC group.

## 3. Results

### 3.1. Participants

**Figure 1:**
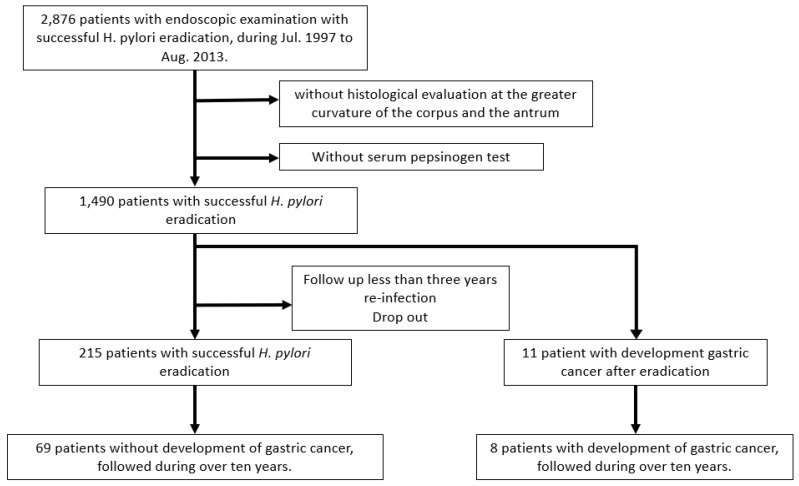
Flow chart of study design.

Of the 2876 enrolled patients, those without histological evaluation at the greater curvature of the corpus and the antrum or without serum pepsinogen testing were excluded. In addition, cases followed-up for less than 3 years, *H. pylori* re-infection cases, and cases with interruption of histological or serological evaluations were excluded. A total of 226 people were ultimately included in this study, and there were 215 cases in the non-GC group and 11 cases in the GC group; 69 cases in the non-GC group and 8 cases in the GC group were followed up for more than 10 years (Figure 1).

### 3.2. Comparison Between GC and Non-GC Groups Before H. pylori Eradication

Table 1 shows the background characteristics at the time of eradication of the GC and non-GC groups. Age at the time of eradication was 57.2 ± 11.4 years in the non-GC group and 65.7 ± 9.8 years in the GC group, significantly higher in the GC group (*p* = 0.008). There was no significant difference in sex. The average observation period after *H. pylori* eradication was 48.9 ± 38.7 months for the non-GC group and 64.0 ± 58.9 months for the GC group (*p* = 0.1712), and the average period between *H. pylori* eradication and development of GC was 49.36 ± 47.37 months.

PG I was 69.6 ± 35.0 ng/mL in the non-GC group and 83.1 ± 87.4 ng/mL in the GC group, and PG II was 21.8 ± 10.7 ng/mL in the non-GC group and 26.0 ± 14.4 ng/mL in the GC group, with no significant differences. PG I/II values were 3.4 ± 1.43 for the non-GC group and 2.6 ± 1.4 for the GC group, significantly lower in the GC group (*p* = 0.037).

The endoscopic gastric atrophic score was significantly higher in the GC group than in the non-GC group (non-GC group 3.4 ± 1.4, GC group 4.3 ± 1.4; *p* = 0.037).

In terms of histological findings, based on the updated Sydney system score, the activity of the greater curvature of the antrum was significantly lower in the GC group than in the non-GC group (GC group 0.50 ± 0.71, non-GC group 1.27 ± 0.74; *p* = 0.004), and inflammation and IM at the greater curvature of the corpus were significantly higher in the GC group than in the non-GC group (inflammation: GC group 2.50 ± 0.53, non-GC group 2.16 ± 0.58; *p* = 0.036; IM: GC group 0.50 ± 0. 71, non-GC group 0.09 ± 0.39; *p* = 0.049).

### 3.3. Changes in Serum Pepsinogen Levels After H. pylori Eradication

Propensity score matching was used to create 10 sex- and age-matched GC and 10 non-GC groups (Table 2). Ratio of serum PG I/II was significantly lower in GC group than in non-GC group (2.6 ± 1.4 vs. 3.97 ± 1.56, *p* = 0.037). Inflammation of the corpus was significantly higher in the GC group than in non-GC group (2.50 ± 0.53 vs. 2.00 ± 0.00, *p* = 0.007). These trends were similar to those before matching.

### 3.4. Changes in Histological Findings After H. pylori Eradication

**Figure 2:**
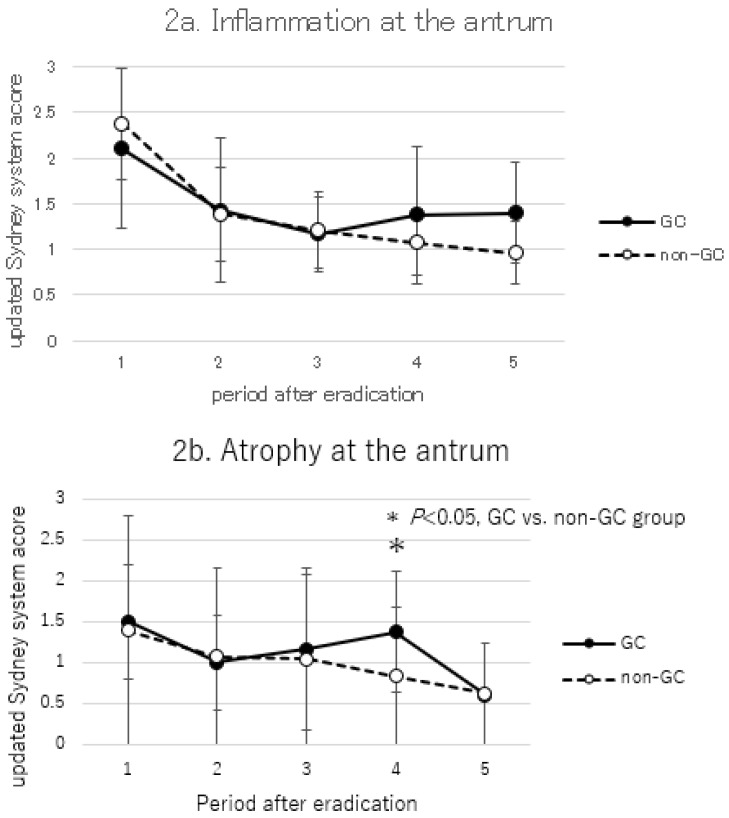

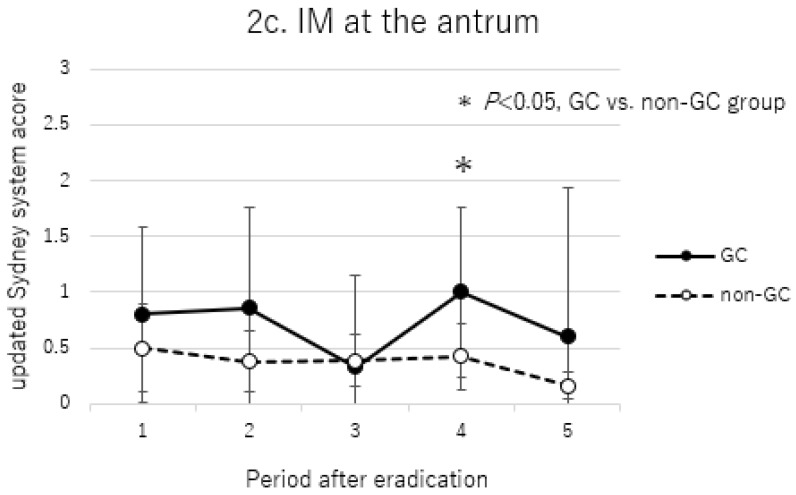
Comparison of updated Sydney system score at the antrum between GC and non-GC group. (**a**) Inflammation at the antrum. (**b**) Atrophy at the antrum. (**c**) IM at the antrum. Periods after eradications were defined as follows: 1, before eradication; 2, 10–14 months after eradication; 3, 15–42 months after eradication; 4, 43–109 months after eradication; 5, more than 110 months after eradication. *p*-values were calculated by Mann–Whitney U test. * *p* < 0.05, GC vs. non-GC group.

**Figure 3:**
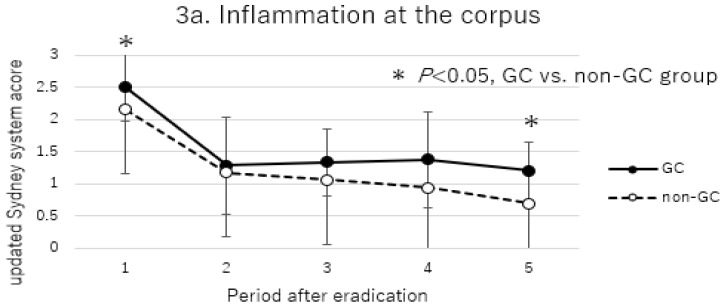

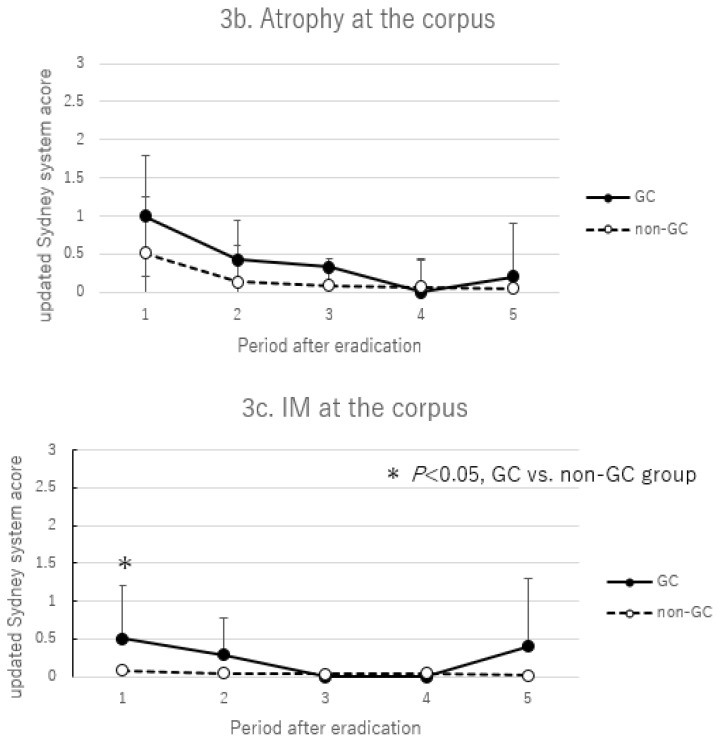
Comparison of updated Sydney system score at the corpus between GC and non-GC group. (**a**) Inflammation at the corpus. (**b**) Atrophy at the corpus. (**c**) IM at the corpus. Periods after eradications were defined as follows: 1, before eradication; 2, 10–14 months after eradication; 3, 15–42 months after eradication; 4, 43–109 months after eradication; 5, more than 110 months after eradication. *p*-values were calculated by Mann–Whitney U test. * *p* < 0.05, GC vs. non-GC group.

Histological changes over time after *H. pylori* eradication in the non-GC group and GC group are shown in Figure 2 and Figure 3 (antrum Figure 2, corpus Figure 3).

Inflammation and atrophy scores were progressively lower in both the antrum and corpus after eradication (inflammation Figure 2a and Figure 3a; atrophy Figure 2b and Figure 3b). In the comparisons between the non-GC and GC groups, inflammation of the corpus was significantly higher before eradication in the GC group, and inflammation remained after long-term follow-up, but atrophy did not show a clear significant difference. IM did not change over time in either the antrum or corpus, and no significant differences were obtained in the comparisons of the non-GC and GC groups (Figure 2c and Figure 3c).

### 3.5. Changes in Endoscopic Atrophic Changes After H. pylori Eradication

**Figure 4:**
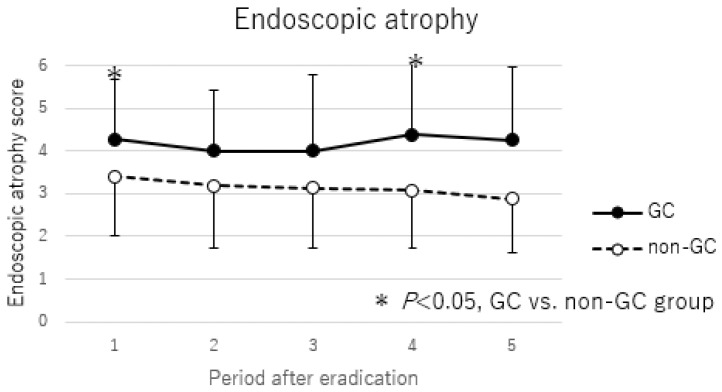
Comparison of endoscopic atrophy score between GC and non-GC group. Periods after eradications were defined as follows: 1, before eradication; 2, 10–14 months after eradication; 3, 15–42 months after eradication; 4, 43–109 months after eradication; 5, more than 110 months after eradication. *p*-values were calculated by Mann–Whitney U test. * *p* < 0.05, GC vs. non-GC group.

Trends in endoscopic atrophic changes after *H. pylori* eradication are shown in Figure 4. Over 10 years after eradication, there was little change in endoscopic atrophic change, although there was some improvement in the non-GC group. In addition, endoscopic atrophic changes were significantly higher in the GC group than in the non-GC group from the time of eradication and remained consistently higher than in the non-GC group after eradication.

### 3.6. Changes in Serum Pepsinogen Levels After H. pylori Eradication

**Figure 5:**
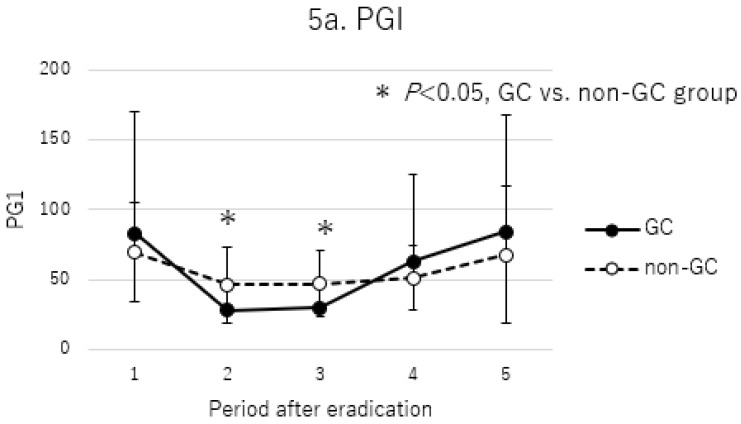

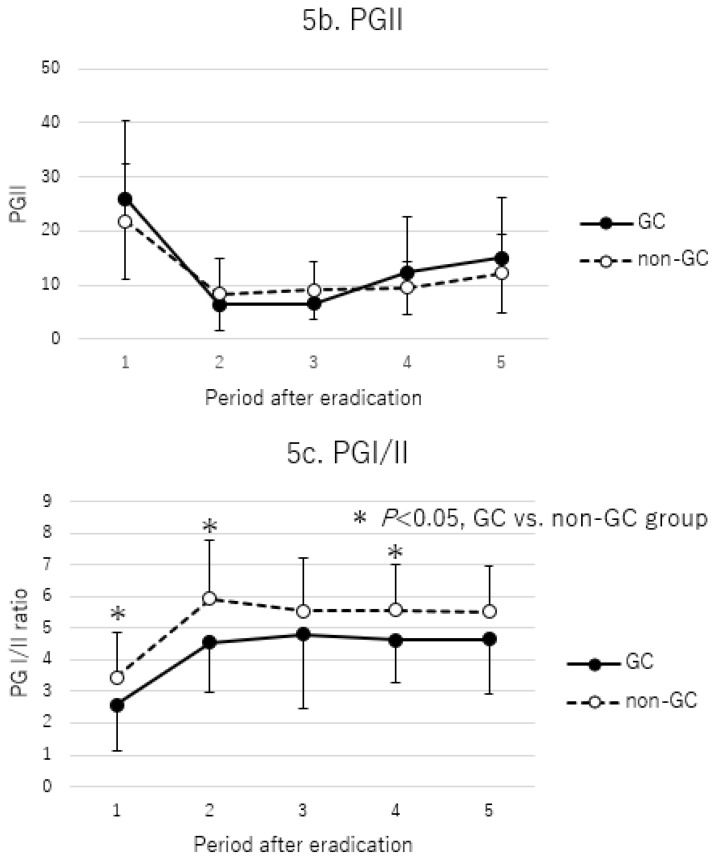
Comparison of serum pepsinogen titer between GC and non-GC group. Periods after eradications were defined as follows: 1, before eradication; 2, 10–14 months after eradication; 3, 15–42 months after eradication; 4, 43–109 months after eradication; 5, more than 110 months after eradication. *p*-values were calculated by Mann–Whitney U test. * *p* < 0.05, GC vs. non-GC group.

Changes in serum PG I and II levels over time after *H. pylori* eradication in the non-GC group and GC group are shown in Figure 5. Both PG I and II levels decreased temporarily until 42 months after eradication and then recovered gradually (Figure 5a,b). In particular, PG I was higher after 110 months than at the time of eradication. For PG I/II, overall, values quickly increased after eradication and remained high (Figure 5c); the GC group maintained lower PG I/II values than the non-GC group during all time periods.

In summary, these results indicate that the GC group consistently had higher endoscopic atrophy scores and lower PG I/II than the non-GC group more than 10 years after *H. pylori* eradication.

## 4. Discussion

With the recent widespread use of *H. pylori* eradication therapy, the proportion of post-eradication GC patients is increasing more rapidly than that of *H. pylori*-infected GC patients in daily practice. In this study, risk factors for the development of GC were investigated by long-term observation of endoscopic, histological, and serological changes in GC and non-GC patients after *H. pylori* eradication.

*H. pylori* eradication therapy has been reported to be effective in preventing GC even after more than 10 years, but it does not completely suppress GC [22]. In the present study, the patients in the non-GC group and the GC group were observed for 204 months and 210 months, respectively. The mean time of occurrence of GC after eradication was 49.36 ± 47.37 months, and the longest time was 150 months, suggesting that follow-up by endoscopic screening for a considerably long period is necessary even after *H. pylori* eradication.

Comparison of the GC and non-GC groups at the time of *H. pylori* eradication showed significant differences in age at *H. pylori* eradication, serum pepsinogen (PG) I/II levels, endoscopic atrophic change, activity at the antrum, inflammation at the corpus, and IM at the corpus on updated Sydney system scoring. These results are generally consistent with previous reports. The older age at the time of eradication suggests a longer duration of *H. pylori* disease. In our previous study, more advanced atrophy was seen in elderly patients with *H. pylori* infection than in younger patients [23], and early *H. pylori* eradication therapy was associated with prevention of GC [24,25]. Take et al. reported that patients with more advanced mucosal atrophy had a higher risk of developing GC after eradication [26,27]. In recent years, efforts have been made to develop a system to prevent GC by *H. pylori* eradication in children and adolescents [28]. Masuyama et al. reported that the incidence of cancer increased with the degree of gastric mucosal atrophy, especially in cases of simultaneous or metachronous carcinogenesis, most of which were cases of advanced atrophy [29]. The results of the present study also confirm that the GC group was older at the time of eradication and had advanced endoscopic mucosal atrophy, so these findings indicate an environment conducive to the development of GC.

On the other hand, IM is a well-known predictor of GC development [2,30]. In particular, the presence of IM in the corpus has been identified as a risk factor for the occurrence of GC after eradication, supporting the aforementioned difference in age at *H. pylori* eradication [31]. Urita et al. investigated the relationship between PG and IM and reported that IM was more common in those with low PG I levels and low PG I/II ratios, and when a PG I/II ratio of 3.0 was used as the cutoff value, IM could be discriminated with sensitivity of 71.7% and specificity of 66.7% [32], suggesting that the IM score was associated with the severity of *H. pylori*-associated gastritis, as well as histological atrophy.

From the above, it is clear that patients with atrophic gastritis or advanced IM at the time of eradication are still at risk for GC even after *H. pylori* eradication therapy.

Regarding histological changes after *H. pylori* eradication, both GC and non-GC groups showed improvement in inflammation and atrophy, but no change in IM. This result is consistent with many meta-analyses and other previous studies [14,15,33,34,35,36,37]. Hwang et al. reported a faster rate of improvement in atrophy than in IM after eradication [36]. Of particular note in the present study is that inflammation and IM were significantly higher in the GC group at the time of eradication, even though long-term observation showed no difference between the two groups. Na et al. showed that gastric mucosal atrophy, IM, OLGA, and OLGIM in a high stage are high-risk factors for GC after *H. pylori* eradication, which is also consistent with the present study results [38]. Therefore, the condition of the gastric mucosa before *H. pylori* eradication may be an important predictor of GC after eradication. In addition, inflammation, atrophy, and IM were irregularly high. Possible reasons for these results include the fact that the GC group already had a higher score than the non-GC group before eradication, but it is not certain. One consideration is that, although the test for the presence of *H. pylori* is not positive, there is a possibility of continued latent infection or re-infection. Fukuda et al. noted that *H. pylori* antibody titers decrease significantly up to 2 years after eradication, but only slowly thereafter [17]. We previously reported that median *H. pylori* antibody titers were significantly higher in the GC group than in the non-GC group from baseline at 0.5–2 years after *H. pylori* eradication [16]. Furthermore, the slow reduction in *H. pylori* antibody titers after *H. pylori* eradication therapy may reflect a delay in the eradication of bacteria that have invaded the parietal cells, lamina propria mucosae, or lymph nodes, and, thus, an increased risk of GC development [39]. The residual gastritis and difficulty improving scores in the GC group may be due to false-negative *H. pylori* infections or *H. pylori* organisms that have penetrated deeply into the gastric parietal cells and lamina propria mucosae.

Unlike histological atrophy, endoscopic mucosal atrophy did not improve in either the GC group or the non-GC group years after *H. pylori* eradication. It has been previously noted that endoscopic atrophy does not improve with eradication [17,40,41]. Endoscopic atrophy is different from mucosal findings before and after eradication, and the atrophic border may not be clear after eradication. However, our previous reports have shown that endoscopic atrophy, histological atrophy, and IM correlate in the antrum even after *H. pylori* eradication [31]. In addition, many previous reports have shown that histological atrophy improves after eradication, but IM is unlikely to improve [14,15,33,34,42]. These considerations suggest that endoscopic atrophy may reflect the presence of IM rather than histological atrophy. We have previously reported that endoscopic atrophy after eradication was more difficult to improve in the GC group than in the non-GC group. This may also suggest that the degree of IM is more severe in the GC group [16]. Endoscopic atrophy and severe IM have been reported to be associated with a high risk of GC after eradication [2,26,29,30]. Although risk factors for GC after eradication at the time of eradication have been reported, there are few reports of risk factors for GC after eradication in the post-eradication gastric mucosa, and only the appearance of map-like redness has been noted as a high-risk factor [43]. The present report also shows that endoscopic atrophy is a predictor of GC risk at any time after eradication, since endoscopic atrophy is higher in the GC group not only before eradication, but also throughout the observation period after eradication.

Serum PG has attracted attention as a factor that predicts the degree of gastric muco-sal atrophy and defines the risk of GC development [20,21], and patients with PG I ≤ 70 ng/mL and PG I/II ≤ 3 are considered a high-risk group for GC. In this study, the mean values of PG I, PG II, and PG I/II were calculated for both groups and compared. The serum PG level is known to fluctuate with *H. pylori* infection status. When *H. pylori* infection causes inflammation of the gastric mucosa, cell disruption occurs, PG I and PG II are released from PG-secreting cells, and serum PG I and PG II levels are temporarily increased. Subsequently, atrophy occurs in the gastric corpus as a result of chronic inflammation, resulting in a decrease in the number of PG-secreting cells and a decrease in serum PG I and PG II levels. However, due to differences in the distribution of PG I and PG II in the stomach, PG II is less affected by atrophy because it is also secreted by the pyloric glands and duodenal glands. Therefore, the more severe the atrophy, the stronger the decrease in PG I, and the PG I/II ratio also decreases with severe gastric mucosal atrophy [44]. Regarding the changes in PG I/II after *H. pylori* eradication, Fukuda et al. reported a 10-year trend after eradication that was similar to the results of the present study [17]. In other words, improvement of inflammation by *H. pylori* reduced the release of PG from PG-secreting cells due to cell disruption, and serum PG levels decreased quickly. Serum PG I/II also increased with improvement of gastric mucosal atrophy. Xia et al. found a significant correlation between serum PG and the degree of histological atrophy; low levels of PG I/II were particularly associated with severe atrophic mucosa [45]. In the present study, comparing the GC and non-GC groups, there was no significant differences in the changes in PG I and PG II, but the PG I/II ratio was always lower in the GC group during the follow-up period. Many previous reports have also indicated that a low PG I/II ratio is a risk factor for GC [11,12,13,20,21,46,47]. The continued low PG I/II ratio in the GC group in the present study may be a useful predictor of risk of post-eradication GC at any time after *H. pylori* eradication, not just before eradication.

In contrast, the PG I/II cutoff values commonly used in the PG method and the PG I/II values obtained in the present study were inconsistent. That is, whereas PG I/II 3.0 is the cutoff value in the ‘ABC method’ [11,21], the mean values of the PG I/II ratio in the GC group were 2.56 ± 1.43 before eradication, 4.53 ± 1.58 10 to 14 months after eradication, 4.80 ± 2.32 15 to 42 months after eradication, 4.61 ± 1.34 43 to 109 months after eradication, and 4.64 ± 1.74 more than 110 months after eradication. Wang et al. measured PG I/II in non-atrophic gastritis, atrophic gastritis, early GC and progressive GC, and they found that it was 9.18 ± 4.10, 7.03 ± 4.55, 3.74 ± 1.40, and 2.05 ± 0.59, respectively. The area under the curve (AUC) was 0.828, and the optimal threshold was 5.04 (sensitivity: 100%; specificity: 70.4%) for the receiver-operating characteristic (ROC) curve of PG I/II at early GC [46]. Considering that PG I/II also increases with recovery of PG I after *H. pylori* eradication, the cutoff value for PG I/II as a risk of GC at any point after eradication may be set higher.

The European Society of Gastrointestinal Endoscopy (ESGE), the European *Helicobacter* Study Group (EHSG), the European Society of Pathology (ESP) and the Sociedade Portuguesa de Endoscopia Digestiva (SPED) have combined efforts to develop evidence-based guidelines on the management of patients with precancerous conditions and lesions of the stomach, and they mention surveillance for gastric cancer corresponding to the degree of progression of gastric mucosal atrophy and IM [48,49]. In this study, the serum PG I/II ratio was found to be a risk factor for the development of gastric cancer in the future, and as mentioned above, this reflects gastric mucosal atrophy and IM, so we believe that this study is in the same direction as the guidelines. Gastrin-17 has recently attracted attention as a new biomarker for predicting GC [50]. A new prediction rule including the PG I/II ratio and the G-17 level has been developed [51], and it is expected to provide a more accurate screening method for GC.

There are several limitations to this study. First, because it was a single-center study, the number of cases of GC in the population that could be observed long-term after eradication was small. Therefore, it was not possible to match patient background and time of onset for the study. Second, it is unclear whether the GC that occurred in these cases actually occurred after *H. pylori* eradication. In the present study, GC detected after *H. pylori* eradication was considered GC after eradication, so some cases were found as early as 6 months after eradication. In general, the doubling time of highly differentiated GC is extremely long [52], and even if a long time has passed after eradication, it is not possible to determine whether it occurred after eradication or whether it was so-called occult cancer. In addition, the histological evaluation of the background mucosa was based on two biopsy specimens from the antrum and gastric corpus, and it has been noted that the score may vary due to differences in the sites from which the specimens are taken. Prospective, large-scale studies are needed to resolve these limitations.

## 5. Conclusions

The patients who develop GC after *H. pylori* eradication are older at the time of eradication, have more histological mucosal injury, and have more severe endoscopic mucosal atrophy. In addition, endoscopic mucosal atrophy and the serum PG I/II ratio are useful predictors of GC in post-*H. pylori*-eradication cases, measured at any given time. If the patients after *H. pylori* eradication come to the hospital, and it is confirmed that they have severe endoscopic mucosal atrophy or low in serum PG I/II, we should instruct them to undergo regular endoscopic surveillance.

## Figures and Tables

**Table 1:** Background of the GC group and non-GC group before *H. pylori* eradication.

	Non-GC Group	GC Group	*p*-Value
Number of cases	215	11	
Gender (Male/Female)	117/98	9/2	0.067 ^a^
Age at *H. pylori* eradication	57.2 ± 11.4 y	65.7 ± 9.8 y	0.008 ^c^
Period between eradication and development of GC (months)		49.36 ± 47.37	
Average of observation period after eradication (month)	48.9 ± 38.7	64.0 ± 58.9	0.171 ^b^
Serum pepsinogen I levels (ng/mL)	69.6 ± 35.0	83.1 ± 87.4	0.31 ^b^
Serum pepsinogen II levels (ng/mL)	21.8 ± 10.7	26.0 ± 14.4	0.177 ^b^
Serum pepsinogen I/II levels	3.4 ± 1.43	2.6 ± 1.4	0.037 ^b^
Endoscopic gastric atrophy score	3.4 ± 1.4	4.3 ± 1.4	0.037 ^c^
Updated Sydney system scoring			
Greater Curvature of the Antrum			
Inflammation	2.38 ± 0.61	2.10 ± 0.88	0.17 ^c^
Activity	1.27 ± 0.74	0.50 ± 0.71	0.004 ^c^
Atrophy	1.40 ± 0.73	1.50 ± 0.71	0.33 ^c^
Intestinal Metaplasia	0.50 ± 0.87	0.80 ± 0.79	0.136 ^c^
Greater Curvature of the Corpus			
Inflammation	2.16 ± 0.58	2.50 ± 0.53	0.036 ^c^
Activity	1.07 ± 0.74	1.00 ± 0.94	0.416 ^c^
Atrophy	0.51 ± 0.75	1.00 ± 1.05	0.089 ^c^
Intestinal Metaplasia	0.09 ± 0.39	0.50 ± 0.71	0.049 ^c^

Data are presented as the mean ± SD. Statistical analyses performed included Fisher’s exact test (a), Student’s *t*-test (b), and the Mann–Whitney U test (c). GC: gastric cancer.

**Table 2:** Comparison of sex- and age-matched gastric cancer and non-gastric cancer groups using propensity score matching.

	Non-GC Group	GC Group	*p*-Value	Matched Non-GC Group	Matched GC Group	*p*-Value
Number of cases	215	11		10	10	
Gender (Male/Female)	117/98	9/2	0.067 ^a^	8/2	8/2	0.45 ^a^
Age at *H. pylori* eradication	57.2 ± 11.4 y	65.7 ± 9.8 y	0.008	65.2 ± 8.52 y	65.7 ± 9.8 y	0.448 ^a^
Serum levels pepsinogen I (ng/mL)	69.6 ± 35.0	83.1 ± 87.4	0.31 ^b^	79.6 ± 31.6	83.1 ± 87.4	0.31 ^b^
Serum levels pepsinogen II (ng/mL)	21.8 ± 10.7	26.0 ± 14.4	0.177 ^b^	21.8 ± 9.22	26.0 ± 14.4	0.177 ^b^
Serum levels pepsinogen I/II	3.4 ± 1.43	2.6 ± 1.4	0.037 ^b^	3.97 ± 1.56	2.6 ± 1.4	0.037 ^b^
Endoscopic gastric atrophy score	3.4 ± 1.4	3.9 ± 1.3	0.147 ^c^	3.7 ± 1.4	3.9 ± 1.3	0.147 ^c^
Updated Sydney system scoring						
Greater Curvature of the Antrum						
Inflammation	2.38 ± 0.61	2.10 ± 0.88	0.17 ^c^	2.40 ± 0.52	2.10 ± 0.88	0.18 ^c^
Activity	1.27 ± 0.74	0.50 ± 0.71	0.004 ^c^	1.00 ± 0.82	0.50 ± 0.71	0.080 ^c^
Atrophy	1.40 ± 0.73	1.50 ± 0.71	0.33 ^c^	1.89 ± 1.05	1.50 ± 0.71	0.18 ^c^
Intestinal Metaplasia	0.50 ± 0.87	0.80 ± 0.79	0.136 ^c^	1.30 ± 1.25	0.80 ± 0.79	0.151 ^c^
Greater Curvature of the Corpus						
Inflammation	2.16 ± 0.58	2.50 ± 0.53	0.036 ^c^	2.00 ± 0.00	2.50 ± 0.53	0.007 ^c^
Activity	1.07 ± 0.74	1.00 ± 0.94	0.416 ^c^	1.22 ± 0.44	1.00 ± 0.94	0.258 ^c^
Atrophy	0.51 ± 0.75	1.00 ± 1.05	0.089 ^c^	0.56 ± 1.01	1.00 ± 1.05	0.181 ^c^
Intestinal Metaplasia	0.09 ± 0.39	0.50 ± 0.71	0.049 ^c^	0.11 ± 0.34	0.50 ± 0.71	0.072 ^c^

Data are presented as the mean ± SD. Statistical analyses performed included Fisher’s exact test (a), the Student *t*-test (b), and the Mann–Whitney U test (c). GC: gastric cancer.

## Data Availability

The dataset used during the current study is available from the corresponding author upon reasonable request.
